# Supplementary material for: Learning curve and surgical outcome of robotic assisted colorectal surgery with ERAS program
Source: Sci Rep. 2022 Nov 29;12:20566. doi: 10.1038/s41598-022-24665-w (PMC9709162; doi:10.1038/s41598-022-24665-w)
Supplement: Supplementary file 2 — Supplementary Information 2. [file 41598_2022_24665_MOESM2_ESM.zip › RAL Raw data-Ñ[▒K/114/OP N_ñw╝╨░O▒Kñσ.pdf]

W83 / W83

臺中榮民總醫院
手術室護理記錄

[redacted]

基本資料

- 室別： 開刀房-17室，手術科別： CRS，手術類別： 常規，日期： 2021/09/25
- 進入時間： 0725 ； 麻醉開始時間： 0801 ； Time Out時間： 0915 ； 劃刀時間： 0916 ； 手術結束時間： 2205 送出時間： 2225 ；
- 麻醉方式： GE

  診斷： Rectal tumor with liver metastasis, cT3N2M1a

  手術名稱： 1.Robot Assisted Low Anterior Resection2.LIVER PARTIAL HEPATECTOMY3.Biliary cholecystectomy4.Laparoscopy

  手術局部用藥： nil

  術前預防性抗生素：
  - 抗生素名稱一： Veterin 1GM IVD/由麻醉護理人員執行
  - 時間： 0855
  - 上次簽章: 歐真勤

  ，追加預防性抗生素：
  - 抗生素名稱一： Veterin 1GM IVD/由麻醉護理師給予
  - 時間： 1255
  - 上次簽章: 陳盈妤
  - 抗生素名稱二： Veterin 1GM IVD/由麻醉護理師給予
  - 時間： 1945
  - 上次簽章: 林湘芸
- 手術醫師： 蔣鋒帆，林志安，陳映菁，謝亦婷
- 麻醉醫師： VS 謝昀叡
-
  - 刷手護士A ： 陳盈妤，時間： 0725，刷手護士B ： 徐敬家，時間： 0725，接替刷手[illegible]： 歐真勤，時間： 1200，接替刷手二： 歐真勤/徐敬家，時間： 1400
  - 流動護士A ： 歐真勤，時間： 0725，接替流動一： 徐敬家，時間： 1130，接替流動二： 陳盈妤，時間： 1400
- 更多接替及流動護士： 接替刷手三： 林湘芸，時間： 1500，接替刷手四： 邱欐嫊，時間： 1730，接替刷手五： 黃鈺雯，時間： 2000，接替流動三： 邱欐嫊，時間： 1500，接替流動四： 黃鈺雯，時間： 1730，接替流動五： 林湘芸，時間： 1730

皮膚狀況

A-V Shunt： 無

術前： 不完整

- 2.瘀傷

部位(術前)：

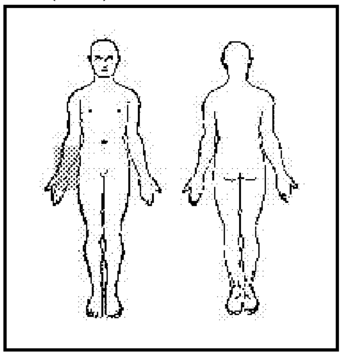

記錄者： 上次簽章: 林湘芸

術後： 不完整

部位(術後)：

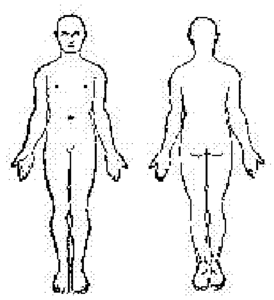

同術前

記錄者： 上次簽章: 林湘芸

手術準備

手術臥式 單一手術臥式

-
    - 手術臥式： 截石臥式
    - 使用附件： 溫毯 38 ℃，頭圈，腳架
    - 皮膚準備： 10%Aq Betadine，Hibiscrub，2% Chlorhexidine
    - 電燒： 使用電燒: 電極片位置： 其它： 導電軟墊，雙極電燒，記錄者： 上次簽章: 歐真勤

止血帶T： 無

植入物

無

標本送檢

- 細菌培養 厭氧，採取部位: 1. GB Bile，數量： 1
- 細胞學 無
- 冰凍切片 部位: 1. L't ureteral，次數： 1
- 病理標本
  組織名稱一：IMA root
  ,
  組織名稱二：distal cut-end
  ,
  組織名稱三：rectum
  ，更多組織名稱
    - 組織名稱四：lateral L.N
    - 組織名稱五：pelvics L.N
    - 組織名稱六：Liver S2
    - 組織名稱七：Liver S4
    - 組織名稱八：Liver S7
    - 組織名稱九：GB
- 截肢 無

標本交付 標本： 9 袋，標本： 0 盒，標本： 0 瓶，細胞學： 0 管，Culture Tube： 1 管，被交付醫師： R 謝亦婷，

- 記錄者： 上次簽章: 林湘芸
- 時間： 2030

傷口術後敷料

傷口敷料： 傷口敷料： 無

- 請於圖上標示部位：

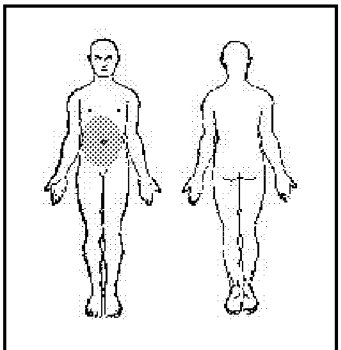

腹部trocar洞

- 傷口敷料： 紗布

導管

導管	天數	導管資訊	插入日期	插入時間	更換日期	拔除日期	拔除時間	拔除原因	預計拔除/更換	預計拔除/更換日期
Foley(1)	1	號碼:16 Fr.; 部位:膀胱;	2021/09/25	0830						
Sump NG Tube(1)	1	號碼:16 Fr.; 深度:60cm; 部位:胃;	2021/09/25	0820						
A-line(1)	1	號碼:20 Ga; 部位:左手;	2021/09/25	0810						
Endotracheal Tube(1)	1	號碼:7.0 mm.; 深度:21.5cm; 部位:氣管;	2021/09/25	0800						
CVC(1)	1	號碼:8 Fr; 深度:15cm; 部位:jugular (右); 象限:RU;	2021/09/25	0820						
Hemo Vac(1)	1	號碼:19 Fr.; 部位:肝下;	2021/09/25	1800						
Hemo Vac(1)	1	號碼:19 Fr.; 部位:骨盆腔;	2021/09/25	1930						
Epidural PCA(1)	1	部位:硬脊膜;	2021/09/25	0755						

用物計數

- 手術中出血量： 450 c.c.
- 手術中尿量： 700 c.c.
-
  - 其它： 無

用物計數：

- 部位一： 大腸部分，1.器械： 正確，2.縫針： 正確，3.刀片： 正確，4.紗布： 正確，5.腹部墊： 未使用，6.紗球： 未使用，7.PEANUTS： 未使用，8.紗條： 未使用，9.絲帶： 未使用，10.針頭： 未使用，11.Vessel loop： 未使用，12.Boots： 未使用，13.Jelco： 未使用，14.棉條： 未使用，
  - 第一次：
    - 刷手護士 ： 歐真勤
    - 流動護士： 上次簽章: 陳盈妤
    - 時間： 1400

  ,
  - 第二次：
    - 刷手護士 ： 歐真勤
    - 流動護士： 上次簽章: 陳盈妤
    - 時間： 1410

  ,
  - 第三次：
    - 刷手護士 ： 歐真勤
    - 流動護士： 上次簽章: 陳盈妤
    - 時間： 1415
- 部位二： Troca，1.器械： 正確，2.縫針： 正確，3.刀片： 正確，4.紗布： 正確，5.腹部墊： 未使用，6.紗球： 未使用，7.PEANUTS： 正確，8.紗條： 未使用，9.絲帶： 正確，10.針頭： 未使用，11.Vessel loop： 未使用，12.Boots： 未使用，13.Jelco： 未使用，14.棉條： 未使用，
  - 第一次：
    - 刷手護士 ： 邱櫂媃
    - 流動護士： 上次簽章: 林湘芸
    - 時間： 1950

  ,
  - 第二次：

- 刷手護士 ： 黃鈺雯
- 流動護士： 上次簽章: 林湘芸
- 時間： 2000

,
- 第三次：
  - 刷手護士 ： 黃鈺雯
  - 流動護士： 上次簽章: 林湘芸
  - 時間： 2030

- 部位三： 造口，1.器械： 正確，2.縫針： 正確，3.刀片： 正確，4.紗布： 正確，5.腹部墊： 未使用，6.紗球： 未使用，7.PEANUTS： 未使用，8.紗條： 未使用，9.絲帶： 未使用，10.針頭： 未使用，11.Vessel loop： 未使用，12.Boots： 未使用，13.Jelco： 未使用，14.棉條： 未使用，
  - 第一次：
    - 刷手護士 ： 黃鈺雯
    - 流動護士： 上次簽章: 林湘芸
    - 時間： 2130

  ,
  - 第二次：
    - 刷手護士 ： 黃鈺雯
    - 流動護士： 上次簽章: 林湘芸
    - 時間： 2140

  ,
  - 第三次：
    - 刷手護士 ： 黃鈺雯
    - 流動護士： 上次簽章: 林湘芸
    - 時間： 2150

備註

備註： 1.davinci 0度&30度 XI scopy:No.2
2.切肝時間:1608
肝下時間:1826
肝重:100g
肝缺血時間:108分14秒
共夾7次

- 記錄者： 上次簽章: 林湘芸
- 時間： 2100

填寫護理人員 林湘芸

正式摘要

	W83 / W83

doctorRank:B
